# A CuO/ZnFe_2_O_4_ heterostructure as an efficient catalyst for NaBH_4_-assisted reduction of chloramphenicol

**DOI:** 10.3762/bjnano.17.97

**Published:** 2026-10-16

**Authors:** Le Thi Hoa, Nguyen Thi Thu Huong, Le Quoc Viet, Trinh Ngoc Dat, Nguyen Duc Cuong, Le Thi Thanh Nhi, Tran Thai Hoa

**Affiliations:** 1 University of Sciences, Hue University, Hue City 530000, Vietnam; 2 The University of Danang, University of Science and Education, Danang City, 550000, Vietnam; 3 University of Education, Hue University, Hue City 530000, Vietnam; 4 Center for Advanced Chemistry, Institute of Research and Development, Duy Tan University, 03 Quang Trung, Danang city, 550000, Vietnam; 5 Faculty of Natural Sciences, Duy Tan University, 03 Quang Trung, Danang city, 550000, Vietnam

**Keywords:** catalysis, chloramphenicol, CuO, NaBH_4_, reduction, ZnFe_2_O_4_

## Abstract

In this study, a CuO/ZnFe_2_O_4_ heterostructure in the form of a flower, consisting of rod-like surface features associated with CuO on ZnFe_2_O_4_ microspheres, was successfully synthesized and evaluated for its catalytic reduction of chloramphenicol (CAP) in the presence of NaBH_4_. Structural and morphological analysis confirmed the successful formation of the heterostructure with enhanced contact between ZnFe_2_O_4_ and CuO. Compared to pure ZnFe_2_O_4_, the CuO/ZnFe_2_O_4_ catalyst exhibited significantly improved catalytic activity for CAP reduction. Reaction conditions strongly influenced reduction efficiency, with optimal performance at pH 6, a catalyst dosage of 2.5 g·L^−1^, and a short reaction time of 20 min. The enhanced catalytic performance under mild, environmentally friendly conditions highlights CuO’s crucial role in promoting surface electron transfer and facilitating reduction. Kinetic analysis at room temperature indicated a change in apparent kinetic behavior during the reaction, with pseudo-first-order behavior in the initial stage and a tendency toward zeroth-order behavior at later times. This evolution is consistent with the involvement of surface-mediated catalytic processes. Overall, the CuO/ZnFe_2_O_4_ heterostructure shows significant potential as an efficient catalyst for rapid CAP reduction under near-neutral conditions, offering a promising strategy for wastewater treatment.

## Introduction

The rapid expansion of agro-industrial sectors, including textile, chemical, fertilizer, pesticide, and pharmaceutical industries, has led to the continuous discharge of large amounts of organic pollutants into aquatic environments. Among these, antibiotic residues are of particular concern due to their persistence and resistance to conventional biological treatment processes. These compounds are often only partially removed, remaining either in treated effluents or temporarily retained in activated sludge, from which they can subsequently be released back into the environment [1]. Chloramphenicol (CAP), a broad-spectrum antibiotic effective against both Gram-positive and Gram-negative bacteria, has been widely used in medical and veterinary applications. However, its presence in water systems poses serious health risks, as CAP has been associated with severe toxic effects in humans, including bone marrow suppression and, in extreme cases, fatal aplastic anemia [2]. Therefore, the effective removal of these contaminants from water bodies is critical.

Various treatment technologies have been developed to address organic pollutants, including membrane separation, biodegradation, adsorption, and catalytic processes. Among these methods, catalytic reduction has attracted considerable attention due to its operational simplicity, cost-effectiveness, high efficiency, and ease of scaling from laboratory studies to practical applications. In recent years, organic pollutants containing nitro groups have received much attention in this field, as the nitro (–NO_2_) functional group can be selectively reduced to less toxic amino derivatives. In this context, sodium borohydride (NaBH_4_) has been widely used as a strong hydrogen donor for reduction conversions [3]. However, in the absence of a catalyst, the reduction process is kinetically unfavorable. Therefore, catalytic surfaces, especially those based on metal nanostructures or metal oxides, play a crucial role in facilitating electron transfer and enabling the efficient delivery of active hydrogen species from BH_4_^−^ to pollutant molecules [4].

Magnetic nanoparticles (MNPs) are one of the magnetic metal nanoparticles widely used in catalysis for environmental treatment. MNPs can be synthesized with different compositions and phases, including pure metals such as Fe, Co, and Ni, metal oxides such as Fe_3_O_4_, γ-Fe_2_O_3_, ferrites such as MFe_2_O_4_ (M = Cu, Ni, Mn, Mg), and metal alloys such as FePt and CoPt [5]. Among them, ZnFe_2_O_4_ is considered a magnetic semiconductor that can act catalytically in the visible-light region because it has a narrow bandgap energy (*E*_g_ = 1.92 eV) with an electric potential (valence band (VB) at 0.38 V, and conduction band (CB) at −1.54 V) [6]. ZnFe_2_O_4_ has a spinel structure with Zn^2+^ occupying tetrahedral positions, and Fe^3+^ ions occupying octahedral positions in the crystal lattice. The exact distribution between cations at these two positions significantly impacts the electronic, magnetic, and catalytic properties of ZnFe_2_O_4_. In general, ZnFe_2_O_4_ has many outstanding characteristics, such as magnetism, optical properties, non-toxicity, and stability in many different environments [7]. However, at the nanoscale, ZnFe_2_O_4_ is often unstable due to its tendency to aggregate and undergo surface oxidation, which can reduce its dispersibility and magnetic performance over time. To overcome these limitations, various surface functionalization and structural modification strategies have been developed to improve stability and dispersibility, thereby enhancing catalytic efficiency. Based on these considerations, the development of efficient and stable catalysts based on ZnFe_2_O_4_ spinel ferrite coupled with transition-metal oxides is a potential approach to overcome existing limitations. Among the latter, CuO is a p-type semiconductor with a narrow bandgap (1.2–2.1 eV), high stability, good conductivity, low cost, and relatively low toxicity [8]; it has proven effective in applications such as energy storage, antimicrobial applications, and photocatalysis [9]. Therefore, the combination of ZnFe_2_O_4_ and CuO is expected to improve catalytic activity by enhancing surface reactivity and promoting electron transfer. In this study, a CuO/ZnFe_2_O_4_ heterostructure was designed and synthesized to enhance the catalytic reduction efficiency of CAP in the presence of NaBH_4_. The influence of key reaction parameters, including pH, catalyst dosage, and reaction time, was systematically investigated to evaluate the efficiency of treating nitro-containing organic compounds.

## Experimental

### Materials

Chloramphenicol (CAP, C_11_H_12_Cl_2_N_2_O_5_), ethylene glycol (C_2_H_6_O_2_), ZnCl_2_, FeCl_3_·6H_2_O, *N*,*N*′-dimethylformamide (DMF), CuCl_2_·2H_2_O, urea (CON_2_H_4_), NaCl, and citric acid (C_6_H_8_O_7_) were purchased from Merck (Germany). No additional purification was performed on the analytical reagents before use, and all experimental procedures used deionized water.

### Synthesis of ZnFe_2_O_4_ NPs

ZnFe_2_O_4_ was synthesized by the hydrothermal method. A mixture of ethylene glycol (60 mL), ZnCl_2_ (0.28 g), FeCl_3_·6H_2_O (1.08 g), urea (2 g), and citric acid (0.5 g) was transferred to a 100 mL Teflon-lined stainless-steel autoclave and heated for 24 h at 160 °C. After cooling to room temperature, the resulting solid was filtered, washed with deionized water, and dried in an oven for 6 h at 60 °C. The resulting solid was transferred to a muffle furnace at 400 °C for 2 h, and the sample was further washed and dried for 6 h at 60 °C [10].

### Synthesis of CuO/ZnFe_2_O_4_

ZnFe_2_O_4_ (600 mg) was added to a solution containing CuCl_2_·2H_2_O (540 mg) and urea (1.0 g) dissolved in DMF (60 mL). The mixture was homogenized using an ultrasonic device for 10 min. Then, this homogeneous solution was transferred to a heat-resistant vessel in an autoclave and heated at 85 °C for 24 h. The product collected by centrifugation (10,000 rpm, 5 min) was washed with DMF and acetone several times. Finally, CuO/ZnFe_2_O_4_ was dried overnight at room temperature.

### Characterization

The crystal structure of the composite materials was analyzed by X-ray diffraction (XRD; Bruker AXS, Germany) using Cu Kα radiation (λ = 1.5406 Å) over the 2θ range of 10–80°. Before analysis, the powder samples were lightly ground and spread evenly on a sample holder to ensure a flat and uniform surface. Surface morphology and microstructure of the samples were examined by scanning electron microscopy (SEM, Hitachi S-4800, Japan). The surface chemical composition and elemental state were studied using X-ray photoelectron spectroscopy (XPS; K-Alpha, Thermo VG, United Kingdom) with monochromated Al Kα radiation as the excitation source. Nitrogen adsorption measurements were performed to evaluate the textural properties of ZnFe_2_O_4_ and CuO/ZnFe_2_O_4_. We calculated the specific surface area using the Brunauer–Emmett–Teller (BET) method and evaluated pore volume and pore width from the adsorption data, including the Barrett–Joyner–Halenda (BJH) analysis.

### Catalytic experiments

In the catalytic reduction experiment, 40 mL of CAP solution (30 mg·L^−1^) at a set pH was mixed with a specified amount of catalyst. Next, 5 mL of NaBH_4_ solution (60 mM) was added, and distilled water was added to reach a total volume of 100 mL. Consequently, the initial CAP concentration (*C*₀) in the reaction system was 12 mg·L^−1^, while the NaBH_4_ concentration was 3 mM. The mixture was stirred at 150 rpm. After every 2 min, the solids were magnetically separated, and 2.5 mL of supernatant was collected for CAP concentration measurement. The removal efficiency was evaluated using the ratio *C*/*C*_0_, decreasing from 1 to 0 as efficiency increased. The effects of time (5–50 min), pH (4–10), catalyst dosage (2.0, 2.5, and 3.0 g·L^−1^), and ionic strength with NaCl concentration (0.1–0.4 M), on removal efficiency were systematically studied to find optimal conditions. All catalytic reduction experiments were conducted under normal laboratory ambient light without light shielding. All experiments were performed under identical ambient-light conditions and with identical reaction-vessel configurations to ensure consistency.

The simulated wastewater matrix was prepared using representative inorganic ions and natural organic matter, as commonly included in synthetic wastewater formulations in previous studies [11–12]. The matrix comprised CAP (12 mg·L^−1^), NaCl (25 mg·L^−1^), NaHCO_3_ (40 mg·L^−1^), Na_2_SO_4_ (25 mg·L^−1^), NaH_2_PO_4_·H_2_O (7 mg·L^−1^), and sodium humate (2 mg·L^−1^). These constituents were chosen to reflect the typical levels of chloride, bicarbonate, sulfate, phosphate, and dissolved organic matter found in complex wastewater matrices. The CuO/ZnFe_2_O_4_ catalyst and NaBH_4_ were then introduced under the same conditions used in the catalytic reduction experiments.

For the recycling experiments, the CuO/ZnFe_2_O_4_ catalyst was magnetically recovered after each catalytic cycle, washed with deionized water, dried, and reused under the same reaction conditions for five consecutive cycles. After each cycle, the separated liquid phase was analyzed by atomic absorption spectroscopy (AAS) to determine the concentrations of dissolved Fe, Cu, and Zn. We further characterized the catalyst recovered after the fifth cycle by XRD and XPS.

## Results and Discussion

### Characterization

**Figure 1:**
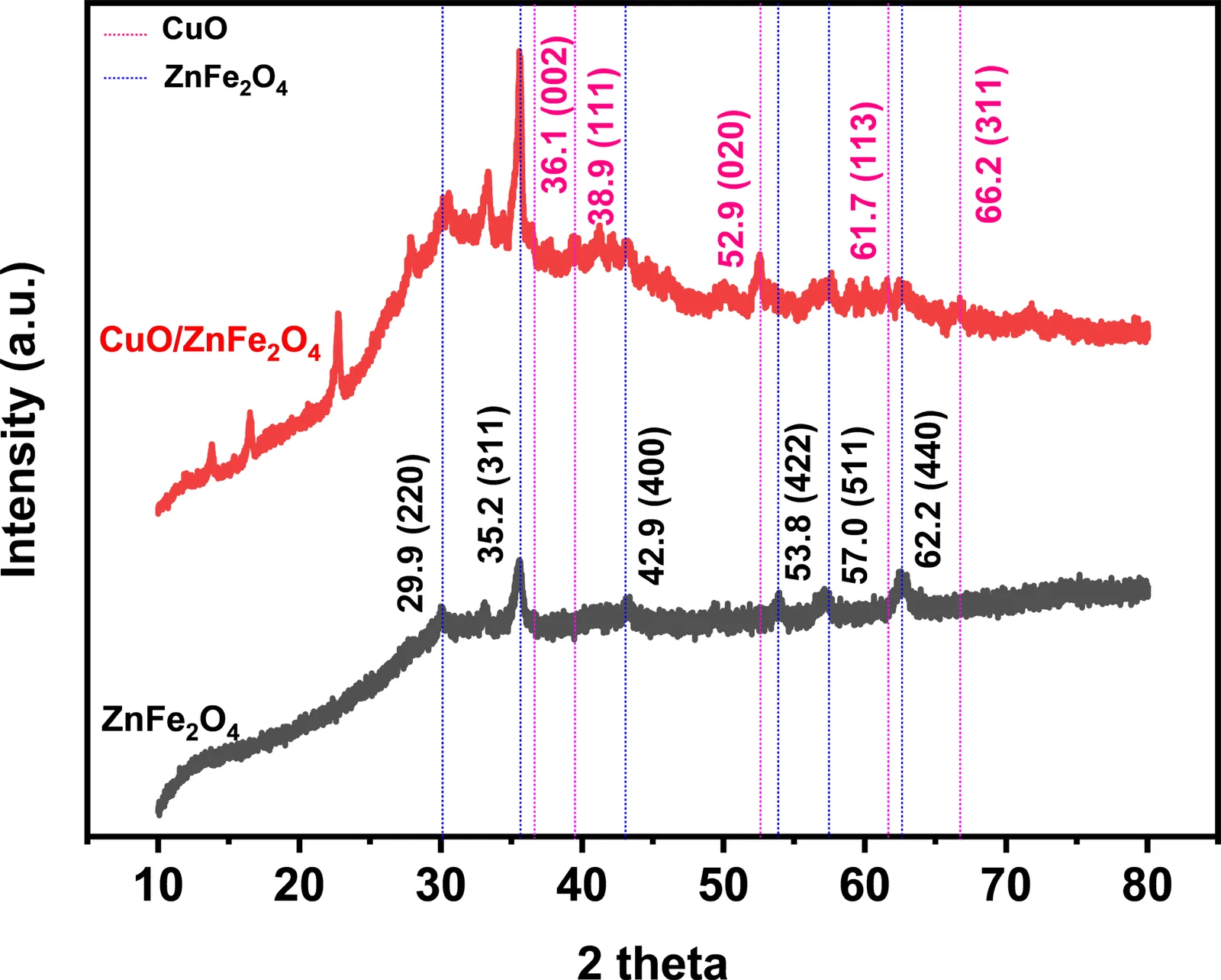
XRD patterns of ZnFe_2_O_4_ and CuO/ZnFe_2_O_4_.

Crystal structure and phase composition of ZnFe_2_O_4_ and CuO/ZnFe_2_O_4_ were studied by X-ray diffraction (XRD). As shown in Figure 1, the diffraction peaks of pure ZnFe_2_O_4_ located at 2θ = 29.9°, 35.2°, 42.9°, 53.8°, 57.0°, and 62.2° can be identified as the (220), (311), (400), (422), (511), and (440) planes of cubic ZnFe_2_O_4_ spinel (PDF#22-1012), indicating the successful formation of well-crystallized ZnFe_2_O_4_ [13–14]. For the CuO/ZnFe_2_O_4_ composite material, all characteristic diffraction peaks of ZnFe_2_O_4_ appear, meaning the spinel structure of ZnFe_2_O_4_ remains intact after the formation of the composite material. Several new diffraction peaks appear at 2θ = 36.1°, 38.9°, 52.9°, 61.7°, and 66.2°, assigned to the planes (002), (111), (020), (113), and (311) of monoclinic CuO (PDF#48-1548), respectively [15–16]. The coexistence of ZnFe_2_O_4_ and CuO diffraction features confirms the successful construction of the CuO/ZnFe_2_O_4_ heterostructure, in which CuO is effectively incorporated without disrupting the ZnFe_2_O_4_ crystal framework.

**Figure 2:**
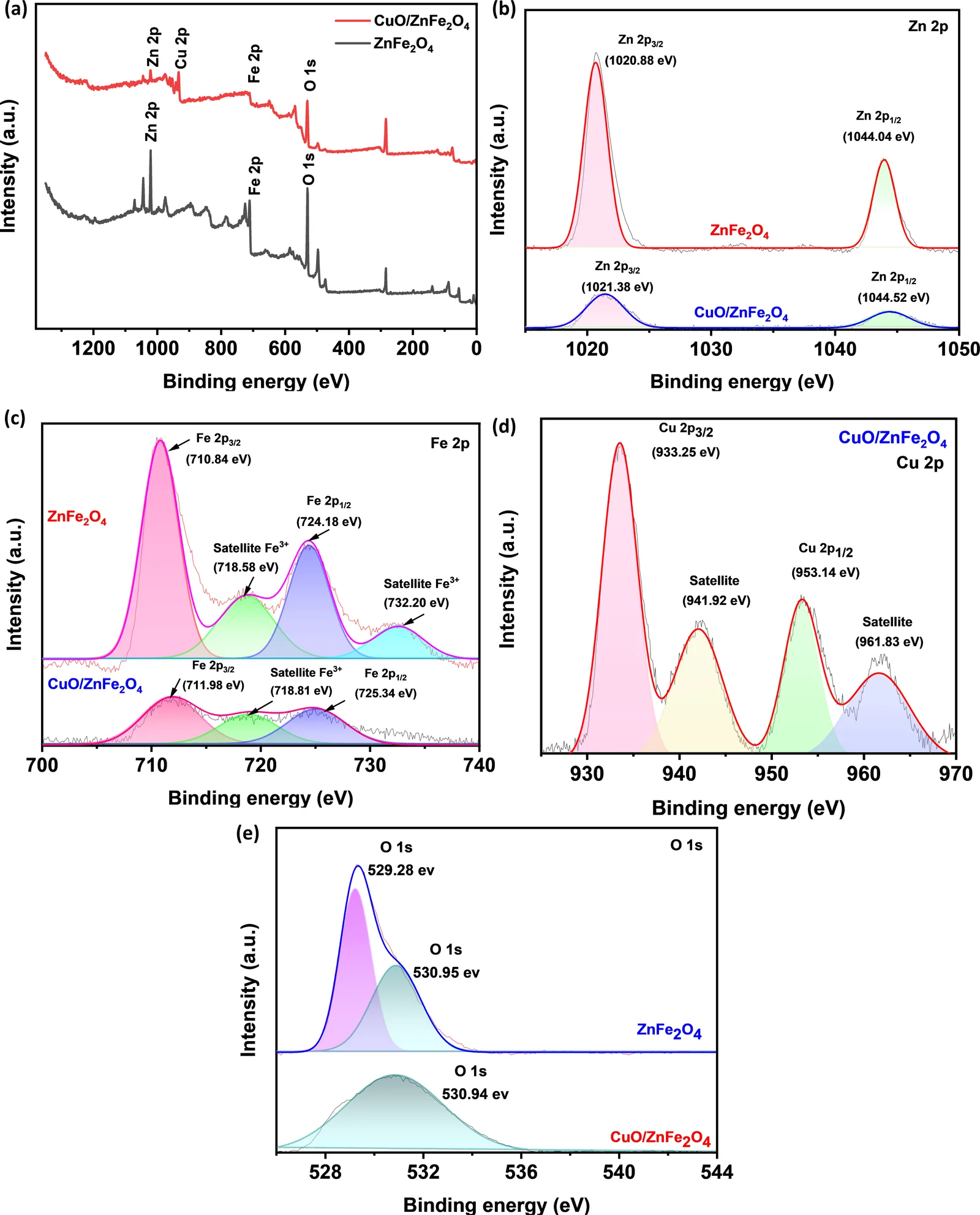
XPS spectra. (a) Survey spectrum, (b) Zn 2p and (c) Fe 2p for ZnFe_2_O_4_ and CuO/ZnFe_2_O_4_, (d) Cu 2p for CuO/ZnFe_2_O_4_ and (e) O 1s for ZnFe_2_O_4_ and CuO/ZnFe_2_O_4_.

While XRD analysis confirmed the phase composition and crystal structure of the ZnFe_2_O_4_ and CuO/ZnFe_2_O_4_ samples, XPS analysis clarified the surface elemental composition, chemical states, and electronic interactions at the interface between ZnFe_2_O_4_ and CuO. The survey XPS spectra of ZnFe_2_O_4_ and CuO/ZnFe_2_O_4_ (Figure 2a) show Zn, Fe, and O in pristine ZnFe_2_O_4_ and, additionally, a clear Cu signal in the heterostructure, confirming the surface incorporation of the Cu-containing component. The high-resolution Zn 2p spectra (Figure 2b) exhibit Zn 2p_3_/_2_ and Zn 2p_1_/_2_ components at approximately 1021 and 1044 eV, respectively, consistent with Zn^2+^ in the spinel ferrite structure [17–18]. The Fe 2p spectra were re-evaluated by considering the characteristic multiplet and shake-up satellite structure of Fe in iron oxides rather than assigning a single unconstrained peak to each formal oxidation state. For ZnFe_2_O_4_, the Fe 2p_3_/_2_ and Fe 2p_1_/_2_ envelopes occur at approximately 710.84 and 724.18 eV, together with satellite features at approximately 718.58 and 732.20 eV (Figure 2c). In CuO/ZnFe_2_O_4_, the corresponding Fe 2p features remain in the Fe(III)-characteristic binding-energy region, with a satellite near 718.81 eV. These spectral features indicate that Fe(III) is the predominant iron oxidation state, consistent with the Zn^2+^Fe_2_^3+^O_4_ spinel structure identified independently by XRD. No distinct additional Fe(I) or Fe(II) components could be reliably resolved from the experimental spectra; therefore, such components were not artificially introduced into the fitting. For Cu 2p (Figure 2d), the main Cu 2p_3_/_2_ and Cu 2p_1_/_2_ peaks are located at 933.25 and 953.14 eV, respectively, with a spin–orbit separation of approximately 19.9 eV. Pronounced shake-up satellite features at approximately 941.92 and 961.83 eV are characteristic of Cu(II) and support CuO as the predominant Cu-containing phase, in agreement with XRD [19]. Accordingly, the Cu 2p analysis was assigned to the experimentally resolved Cu(II) doublet and its characteristic shake-up satellite contributions. Distinct Cu(I) or Cu(III) components were not resolved and were therefore not introduced artificially into the deconvolution. The O 1s spectra (Figure 2e) were interpreted according to oxygen chemical environments rather than formal metal oxidation states. In ZnFe_2_O_4_, the lower-binding-energy contribution at approximately 529.28 eV is assigned primarily to lattice O^2−^ in metal–oxygen bonds, whereas the higher-binding-energy contribution near 530.94 eV is associated with surface/defect-related and hydroxy/adsorbed oxygen species [17]. In the CuO/ZnFe_2_O_4_ heterostructure, the broadened O 1s envelope reflects overlapping oxygen environments associated with the ZnFe_2_O_4_ and CuO lattices and surface-related oxygen species. Because these contributions overlap strongly, the O 1s spectrum is discussed in terms of chemically plausible oxygen environments without overassigning individual components. Overall, the revised XPS analysis supports Zn^2+^, predominantly Fe^3+^, and predominantly Cu^2+^ as the resolved cationic states in the CuO/ZnFe_2_O_4_ heterostructure, consistent with the ZnFe_2_O_4_ and CuO phases independently identified by [17–22].

**Figure 3:**
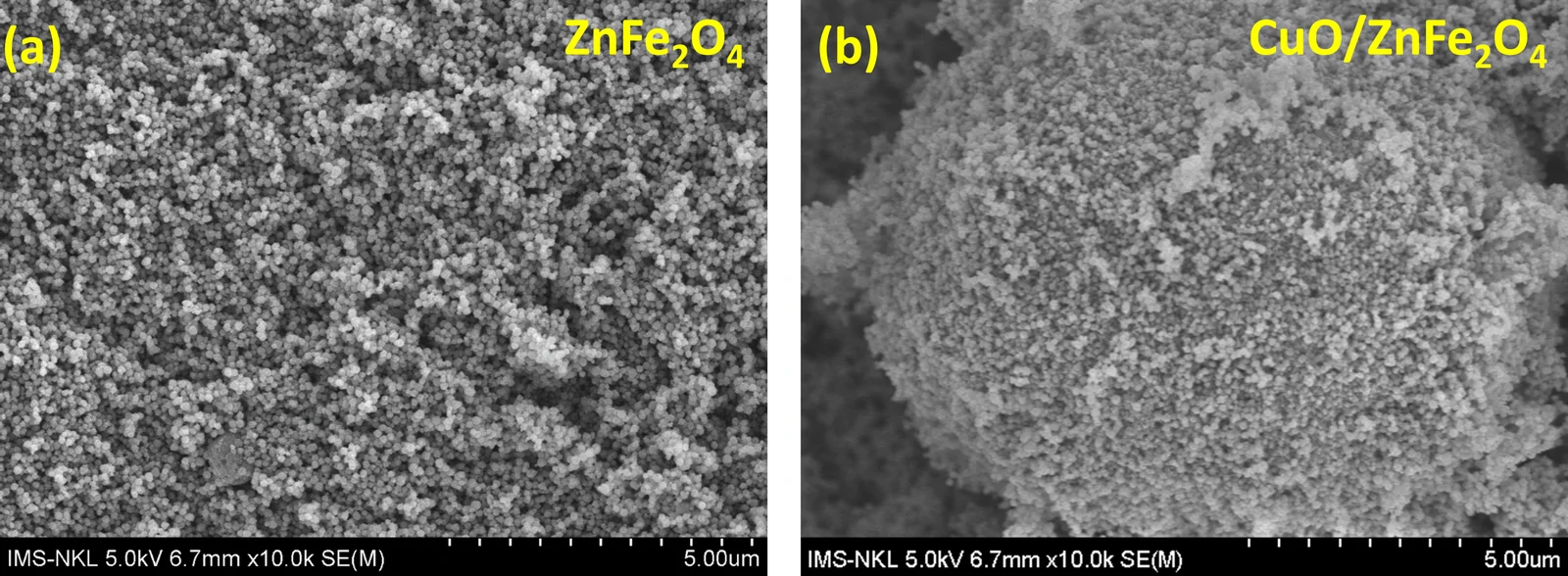
The SEM images of ZnFe_2_O_4_ (a) and CuO/ZnFe_2_O_4_ (b). The scale bar is 5 μm for both images.

N_2_ adsorption measurements were performed to assess the textural properties of ZnFe_2_O_4_ and CuO/ZnFe_2_O_4_. The BET specific surface area decreased from 56.89 m^2^·g^−1^ for ZnFe_2_O_4_ to 49.27 m^2^·g^−1^ for CuO/ZnFe_2_O_4_, showing that CuO incorporation did not enlarge the specific surface area. In contrast, the total pore volume increased from 0.1653 to 0.1825 cm^3^·g^−1^, while the average pore width (4V/A by BET) increased from 11.62 to 14.82 nm. The BJH adsorption average pore width likewise increased from 11.06 to 13.45 nm. These results indicate that CuO incorporation modifies the pore architecture of ZnFe_2_O_4_ rather than simply increasing its specific surface area. The moderate decrease in BET surface area may be associated with partial coverage or blockage of accessible ZnFe_2_O_4_ surface sites by the deposited CuO component, whereas the increased pore volume and average pore width suggest larger accessible pore/interparticle void dimensions in the heterostructure. While XPS provided strong evidence for the chemical states and confirmed the successful formation of the CuO/ZnFe_2_O_4_ composite, SEM was employed to further elucidate the surface morphology and assess the spatial distribution of the CuO phase on the ZnFe_2_O_4_ matrix. As illustrated in Figure 3, the CuO/ZnFe_2_O_4_ material exhibits flower-like hierarchical microstructures with characteristic dimensions of approximately 4 µm, as estimated from the SEM images. These structures are formed by the assembly of densely packed rod-like surface features radiating outward, resulting in a rough and highly textured morphology. Compared with ZnFe_2_O_4_ (inset), which displays a relatively compact, granular surface, the incorporation of CuO significantly alters the surface architecture, increasing surface roughness. Such hierarchical heterostructures are expected to provide more accessible active sites and facilitate interfacial electron transfer [23] during catalytic reaction.

### Catalytic activity

**Figure 4:**
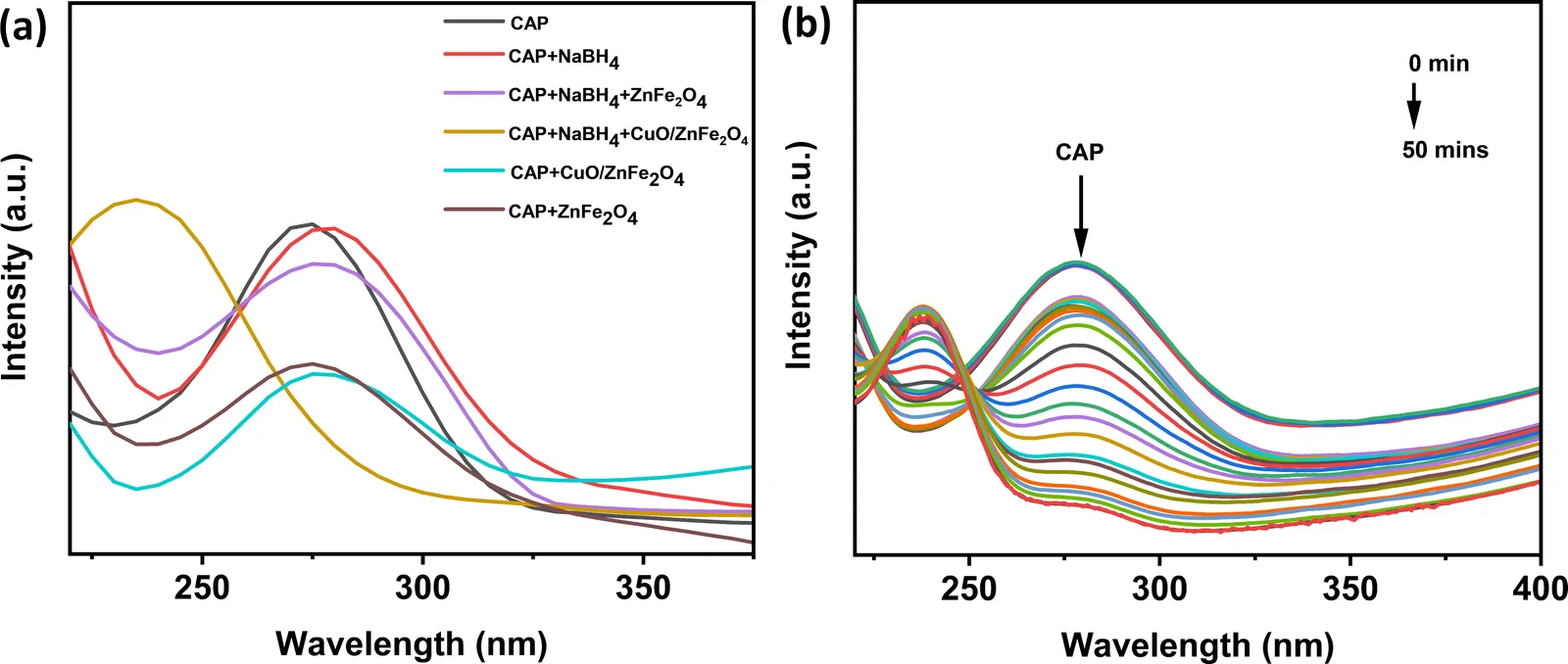
UV–vis spectra of the CAP solution before and after the removal processes (a) all samples; (b) CuO/ZnFe_2_O_4_ over 50 min of reaction time.

The catalytic reduction of CAP was examined using NaBH_4_ as the electron donor and both ZnFe_2_O_4_ and CuO/ZnFe_2_O_4_ as heterogeneous catalysts. The reaction progress was monitored by observing UV–vis spectral changes of CAP under various conditions: CAP alone, CAP with NaBH_4_, CAP with ZnFe_2_O_4_, CAP with CuO/ZnFe_2_O_4_, CAP with NaBH_4_ and ZnFe_2_O_4_, and CAP with NaBH_4_ and CuO/ZnFe_2_O_4_, as illustrated in Figure 4a. These control experiments were performed to assess the possible contribution of adsorption and to distinguish it from the NaBH_4_-assisted catalytic conversion of CAP. In the absence of NaBH_4_, the absorption intensity of CAP remained nearly constant in the presence of both ZnFe_2_O_4_ and CuO/ZnFe_2_O_4_, indicating that adsorption alone contributed minimally to CAP removal under the tested conditions. Similarly, NaBH_4_ alone did not significantly alter the absorption spectrum of CAP, demonstrating the kinetic limitation of direct reduction. When ZnFe_2_O_4_ was combined with NaBH_4_, only a slight decrease in CAP absorption intensity at approximately 280 nm was observed, without the emergence of a new absorption band, suggesting limited catalytic conversion. In contrast, the CuO/ZnFe_2_O_4_-NaBH_4_ system showed a substantial decrease in the characteristic CAP absorption band at approximately 280 nm, along with the appearance and growth of a new absorption feature at around 236 nm. These spectral changes suggest that the enhanced CAP removal in the CuO/ZnFe_2_O_4_-NaBH_4_ system is primarily due to catalytic transformation promoted by NaBH_4_, rather than adsorption. The improved catalytic performance is likely related to the incorporation of CuO, which may facilitate interfacial electron transfer and promote CAP conversion, while ZnFe_2_O_4_ provides a supporting surface and contributes to the overall catalytic process. Although the detailed reaction pathway remains to be elucidated, the comparative results in Figure 4a demonstrate that the CuO/ZnFe_2_O_4_ composite plays a more significant role in the NaBH_4_-assisted conversion of CAP than ZnFe_2_O_4_ [24–25]. The kinetic change of the reduction process is more clearly shown in Figure 4b, which depicts the decrease in the absorption intensity of CAP at 280 nm over reaction time after the addition of CuO/ZnFe_2_O_4_ in the presence of NaBH_4_. The absorption intensity decreases continuously throughout the reaction period, indicating that CAP is efficiently converted under these catalytic conditions. These results confirm that the CuO/ZnFe_2_O_4_ system exhibits superior catalytic activity compared to ZnFe_2_O_4_ alone and highlight the role of CuO in enhancing the CAP reduction efficiency in the NaBH_4_ system.

**Figure 5:**
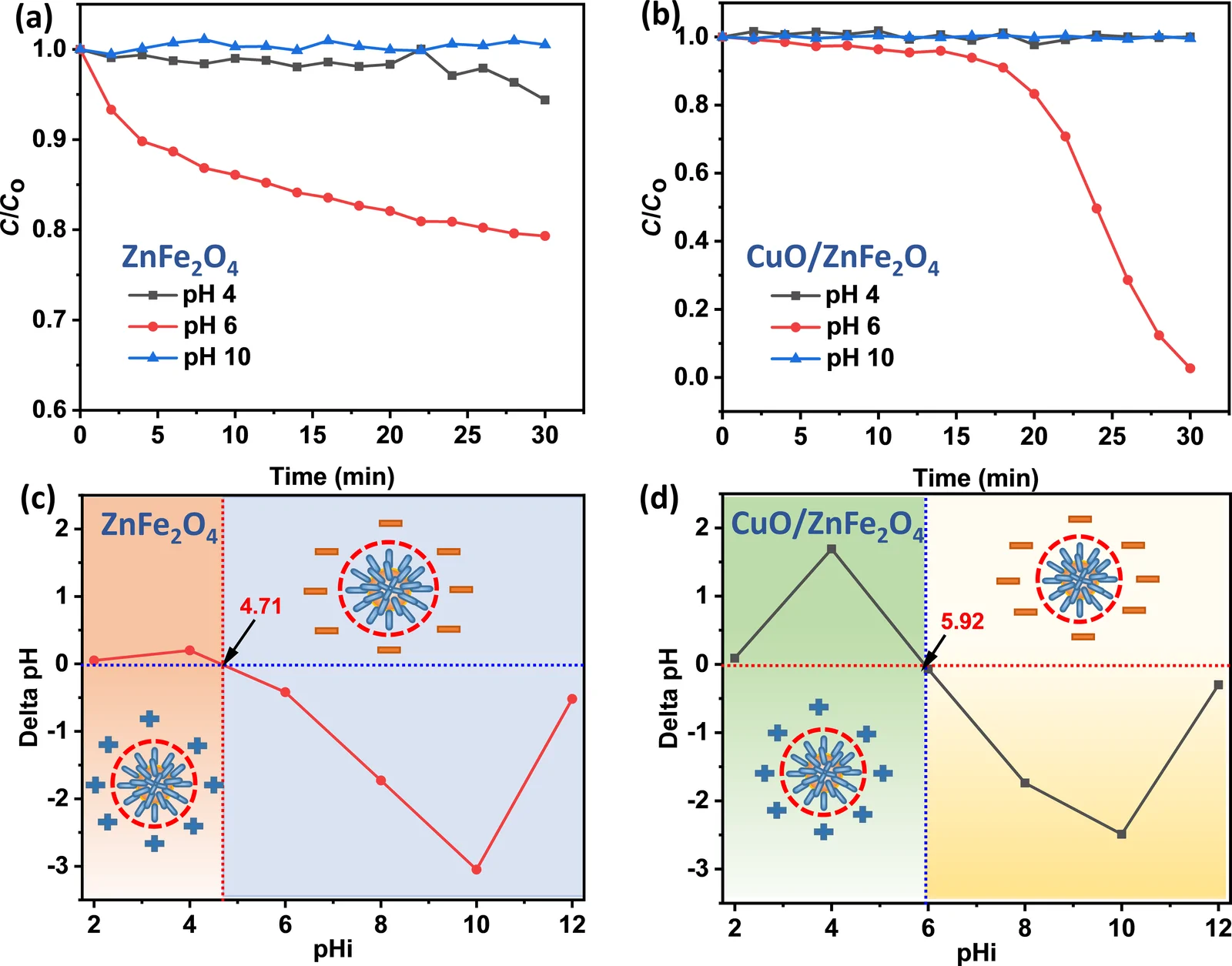
(a, b) Effect of pH on the reduction of CAP; pH_PZC_ of (c) ZnFe_2_O_4_ and (d) CuO/ZnFe_2_O_4_.

The results of the pH effect survey (Figure 5) show that catalytic reduction activity strongly depends on the reaction environment and the material’s surface. For ZnFe_2_O_4_, the absorption spectrum changed only slightly over time within the investigated pH range (pH 4, 6, and 10), indicating limited CAP removal under these conditions. Together with the control experiments in Figure 4a, which showed minimal adsorption in the absence of NaBH_4_, these results indicate that pristine ZnFe_2_O_4_ exhibits limited catalytic activity in the NaBH_4_-assisted system compared with the CuO/ZnFe_2_O_4_ heterostructure [26]. Conversely, the CuO/ZnFe_2_O_4_ system exhibits a significant pH dependence, with the highest catalytic performance observed at pH 6. Under this condition, the *C*/*C*₀ ratio decreases markedly, indicating rapid CAP conversion. The enhanced activity near pH 6 may reflect a favorable balance between catalyst surface charge, CAP–surface interactions, and the availability of BH_4_^−^-derived reducing species, thereby facilitating interfacial electron transfer [27]. At more acidic or alkaline pH, less favorable surface interactions may reduce the catalytic performance. Furthermore, the incorporation of CuO provides redox-active surface sites that may facilitate electron transfer [28], which may explain the enhanced catalytic performance of CuO/ZnFe_2_O_4_ compared with ZnFe_2_O_4_.

**Figure 6:**
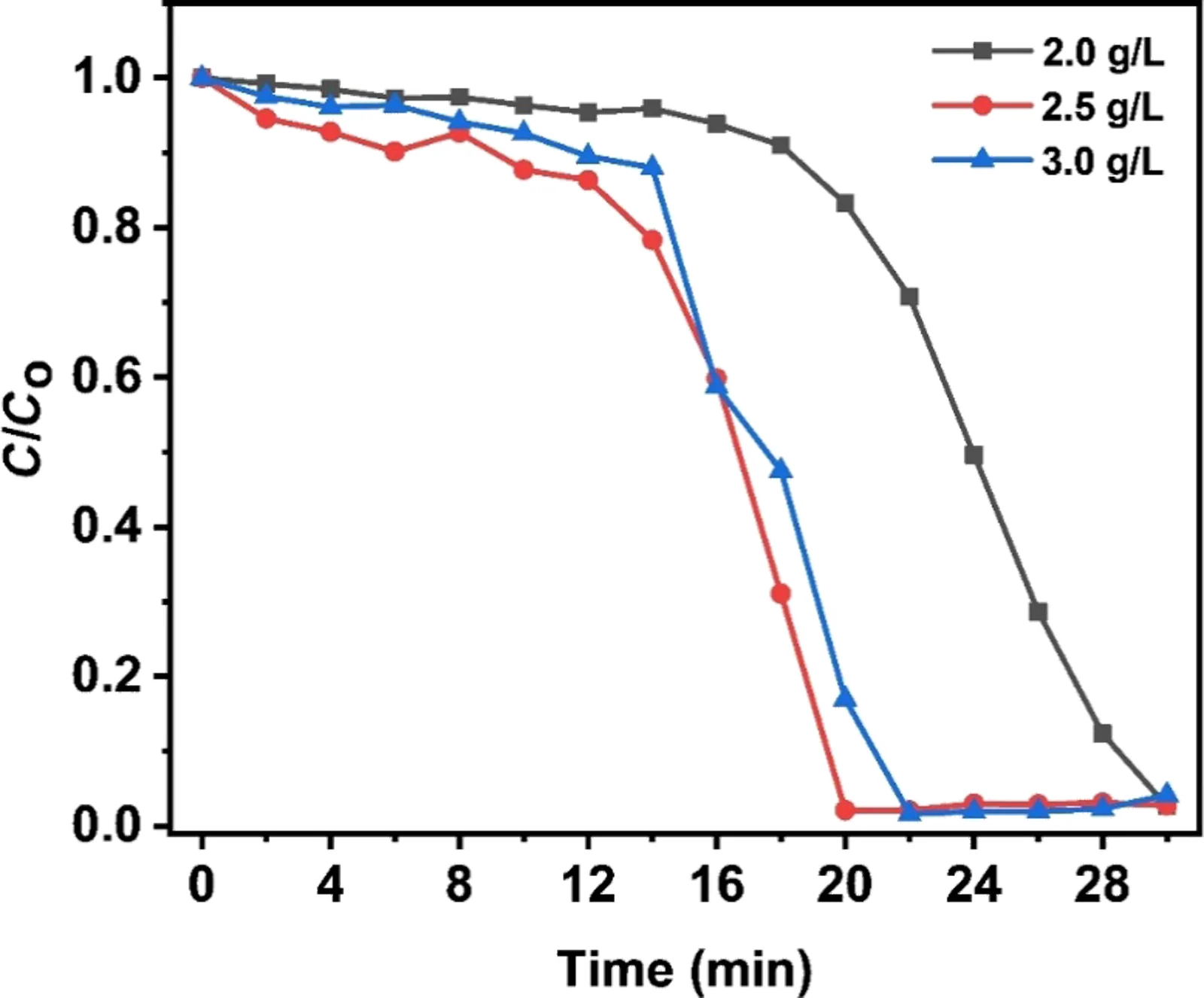
Effect of catalyst dosage on the catalytic reduction of CAP using CuO/ZnFe_2_O_4_ at catalyst dosages of 2.0, 2.5, and 3.0 g·L^−1^.

Figure 6 illustrates the effect of catalyst dosage (2.0, 2.5, and 3.0 g·L^−1^) on the catalytic removal efficiency of CAP in the presence of NaBH_4_. Compared with the dosage of 2.0 g·L^−1^, increasing the CuO/ZnFe_2_O_4_ loading to 2.5 and 3.0 g·L^−1^ significantly enhanced both the removal efficiency and the reaction rate throughout the catalytic process. Specifically, the system containing 2.0 g·L^−1^ of catalyst required approximately 30 min to achieve high CAP removal efficiency, whereas the reaction time was shortened to 20 min and 22 min for catalyst dosages of 2.5 and 3.0 g·L^−1^, respectively. The improved catalytic performance at higher catalyst loadings can be attributed primarily to the increased availability of CuO-containing active sites, which facilitate interactions among BH_4_^−^, CAP, and the catalyst surface and promote interfacial electron transfer [28]. However, when the catalyst dosage exceeded 2.5 g·L^−1^, the catalytic activity enhancement became insignificant and even declined slightly. This behavior is likely associated with the limited amount of NaBH_4_ in the reaction system, which restricts the generation of active hydrogen species and the transfer of electrons required for the reduction reaction [29]. Therefore, a catalyst dosage of 2.5 g·L^−1^ was selected as the optimal condition for subsequent experiments.

**Figure 7:**
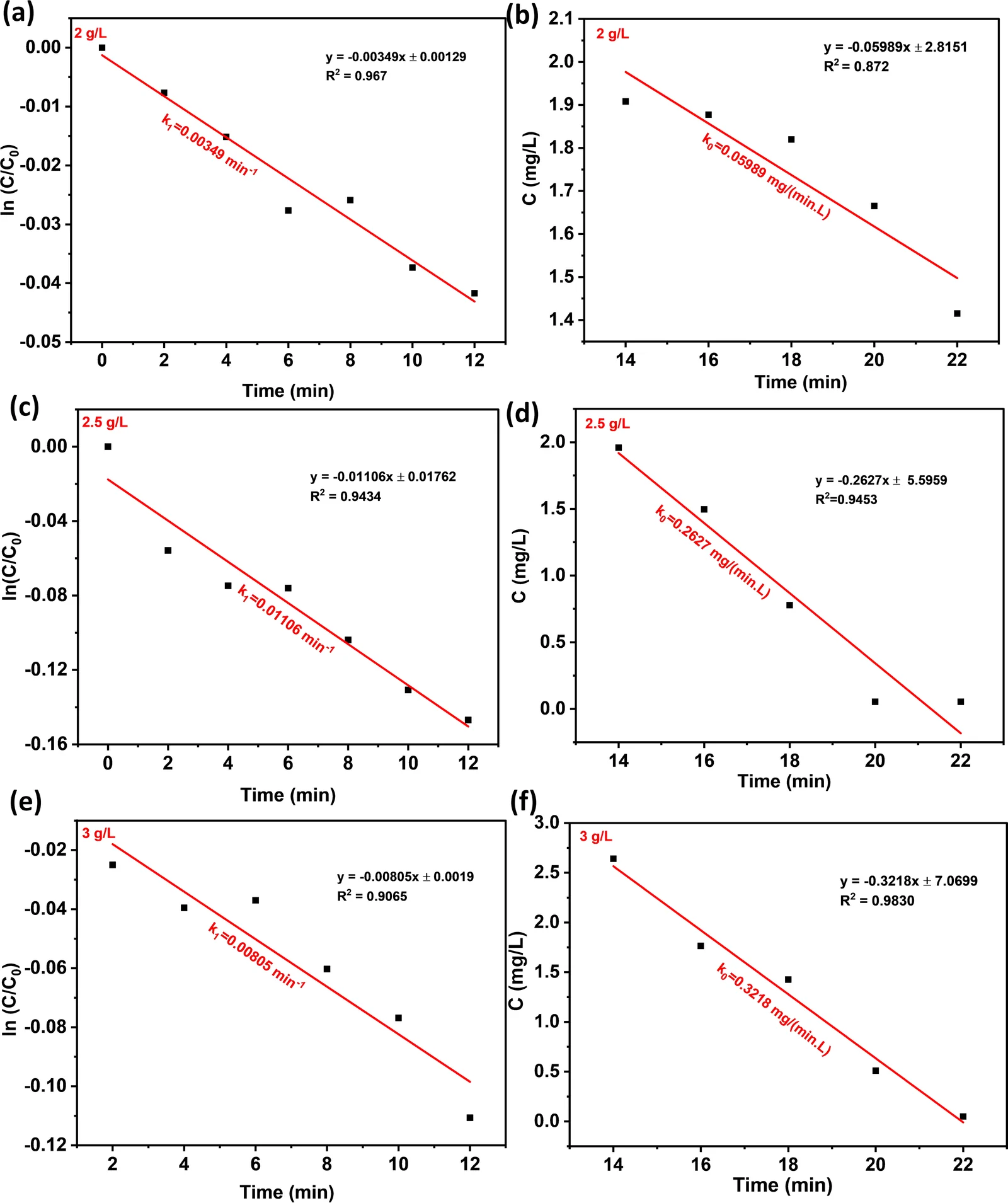
Kinetic analysis of CAP reduction of CuO/ZnFe_2_O_4_ at different dose of 2 g/L (a, b), 2.5 g/L (c, d) and 3 g/L (e, f) at room temperature.

Kinetic analysis results (Figure 7) at catalyst dosages of 2.0, 2.5, and 3.0 g·L^−1^ at room temperature indicate that the CAP reduction on CuO/ZnFe_2_O_4_ cannot be adequately interpreted as a single kinetic regime over the entire reaction period. The transition between the two apparent kinetic regions was assigned from the observed change in linearity of the kinetic profiles, which occurred after approximately 12–14 min. Accordingly, the 0–12 min interval was used to describe the initial apparent regime and the 14–22 min interval to evaluate the later regime. For 0–12 min, the ln(*C*/*C*₀) versus time plots showed good linearity with the pseudo-first-order model (Figure 7a,c,e). At later times, the profiles deviated from the initial pseudo-first-order behavior and showed a tendency toward zeroth-order behavior (Figure 7b,d,f). These kinetic assignments should therefore be regarded as apparent descriptions of different portions of the reaction profile rather than as proof of a sharp mechanistic transition at a single time point. The apparent rate constants also showed a non-monotonic dependence on catalyst dosage. In particular, *k*_1_ decreased from 0.01106 min^−1^ at 2.5 g·L^−1^ to 0.00805 min^−1^ at 3.0 g·L^−1^, whereas the later-stage *k*₀ increased from 0.2627 to 0.3218 mg·L^−1^·min^−1^. This difference is not a direct contradiction because *k*_1_ and *k*₀ describe different apparent kinetic regions and have different dimensions; they should therefore not be compared as a single dose-dependent rate parameter. The results instead indicate that increasing the nominal catalyst dosage beyond 2.5 g·L^−1^ does not produce a proportional improvement in the apparent reaction rate in all stages of the process, consistent with the small decrease in overall catalytic performance observed at 3.0 g·L^−1^. The kinetic profiles presented here were obtained from individual experimental runs rather than replicate kinetic measurements; therefore, statistically meaningful standard deviations and error bars cannot be calculated retrospectively. The kinetic interpretation is consequently limited to the measured profiles and their apparent fitting behavior, without assigning statistical significance to differences between fitted parameters. The observed evolution in kinetic behavior may reflect changes in surface coverage by CAP, reaction intermediates, BH_4_^−^-derived species, or borate products as the reaction proceeds. Such behavior is qualitatively consistent with a surface-mediated Langmuir–Hinshelwood-type process, but the present kinetic data alone do not establish a unique mechanistic model [30–31]. Among the investigated catalyst dosages, 2.5 g·L^−1^ provided the best overall CAP removal performance within 20 min and was therefore selected as the optimum dosage.

**Figure 8:**
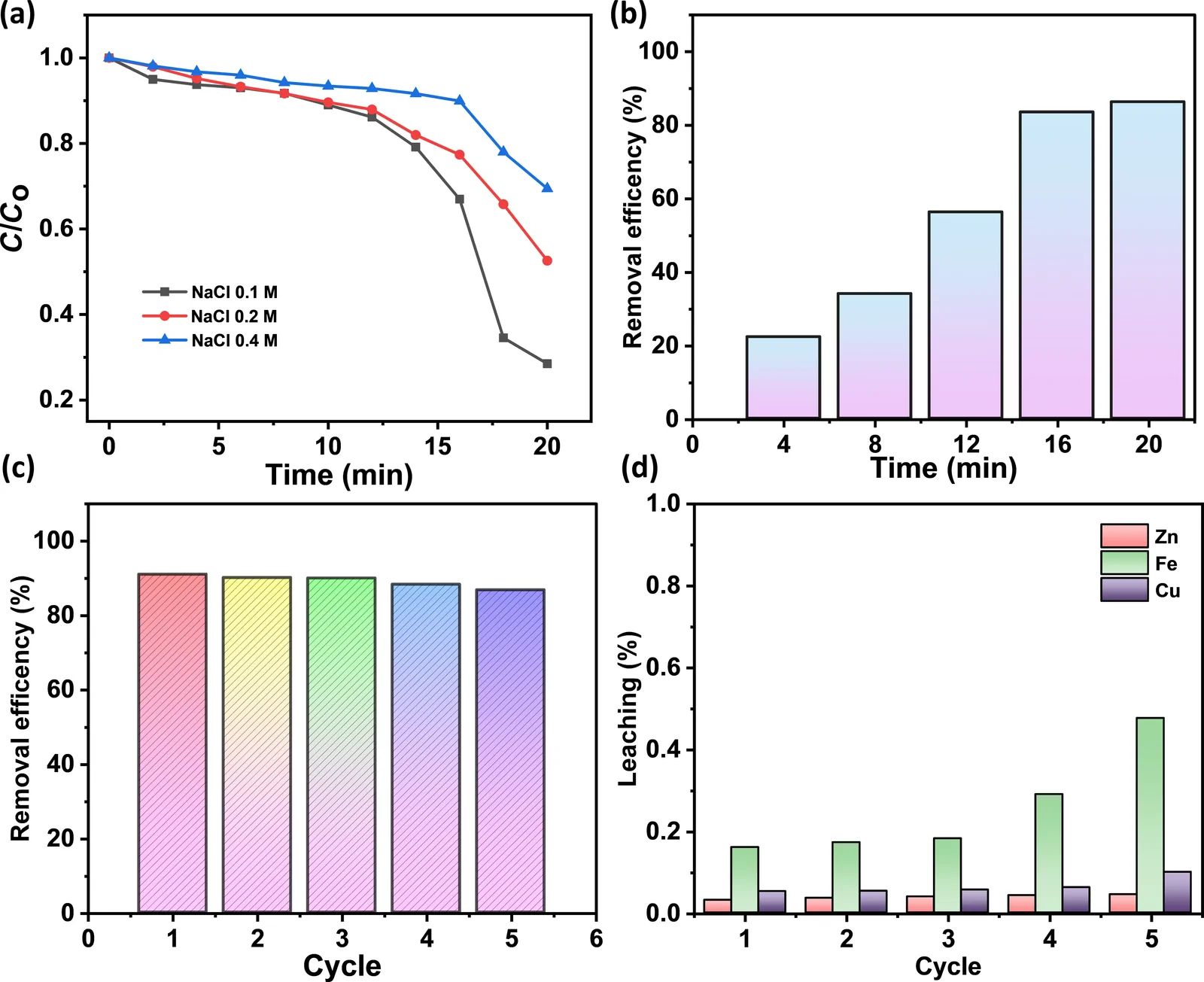
(a) Effect of ionic strength on CAP removal, (b) CAP removal efficiency in simulated wastewater, and (c) recyclability of CuO/ZnFe_2_O_4_ over five catalytic cycles.

Different concentrations of NaCl were added to the CAP solution to evaluate the effect of ionic strength on the catalytic activity of CuO/ZnFe_2_O_4_. As shown in Figure 8a, both the decomposition efficiency and the rate of CAP reduction were strongly affected by ionic strength. As the NaCl concentration increased from 0.1 to 0.4 M, the reduction process became slower, requiring a longer reaction time to achieve equivalent removal efficiency. At relatively low ionic strengths, the electrolyte had only a minor effect on the catalytic reduction of CAP; however, a pronounced inhibitory effect was observed at higher ionic strengths, especially at 0.4 M. This phenomenon suggests that excess ions in solution may hinder the interaction among BH_4_^−^, CAP, and the CuO/ZnFe_2_O_4_ surface, thus inhibiting adsorption and electron transfer through the surface during catalysis, affecting catalytic efficiency [32]. Overall, these results indicate that the CuO/ZnFe_2_O_4_ heterostructure exhibits more favorable catalytic performance under conditions of low ionic strength. To further evaluate the performance of CuO/ZnFe_2_O_4_ in a more complex aqueous environment, a simulated wastewater system containing typical inorganic salts and sodium humate, as commonly used in synthetic wastewater formulations reported in previous studies [11–12], was investigated. Under simulated wastewater conditions, the CAP removal efficiency (Figure 8b) decreased relative to that observed in deionized water, yet remained notably high at 86.25%. The observed reduction aligns with the inhibitory trend observed in experiments on NaCl ionic strength (Figure 8a), although the current experiments could not isolate the individual contributions of each component in the simulated wastewater. Nevertheless, maintaining a CAP removal efficiency of 86.25% in the simulated wastewater environment demonstrates that the CuO/ZnFe_2_O_4_–NaBH_4_ system retains significant catalytic activity under more complex water conditions.

**Figure 9:**
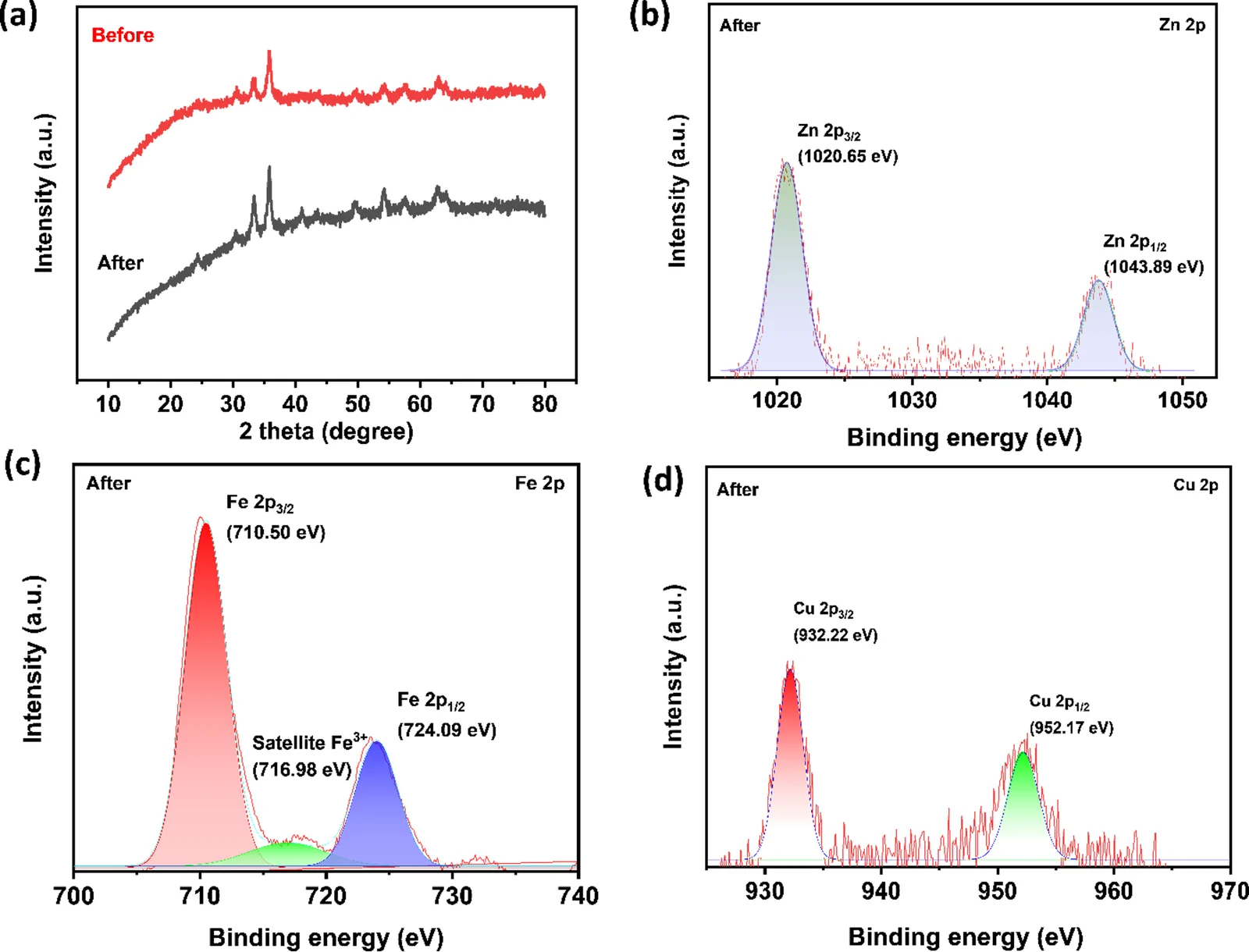
Post-reaction characterization of CuO/ZnFe_2_O_4_ recovered after five catalytic cycles: (a) XRD pattern, (b) Zn 2p, (c) Fe 2p, and (d) Cu 2p high-resolution XPS spectra.

The reusability of the CuO/ZnFe_2_O_4_ catalyst for CAP reduction in the presence of NaBH_4_ was evaluated over five consecutive reaction cycles. As shown in Figure 8c, the CAP removal efficiency decreased only modestly from 91.0% in the first cycle to 86.8% after the fifth cycle, indicating that the catalyst retained substantial catalytic activity during repeated use. The modest performance loss may reflect a combination of catalyst loss during recovery, surface modification, accumulation of surface-bound species, and metal leaching during repeated operation. AAS analysis (Supporting Information File 1, Table S1) of the liquid phase after each catalytic cycle confirmed measurable Fe, Cu, and Zn leaching. These results indicate that, although the catalyst retains substantial catalytic activity over five cycles, metal release occurs under the investigated reaction conditions and limits the present catalyst system. To further assess the catalyst after repeated use, the material recovered after the fifth catalytic cycle was characterized by XRD and XPS (Figure 9).

After five consecutive catalytic cycles, the XRD pattern of the recovered catalyst still showed the main diffraction features of the CuO/ZnFe_2_O_4_ heterostructure (Figure 9a). This result indicates that the major crystalline framework remained detectable after repeated use. The XPS spectra taken after the reaction provided more information about the catalyst’s surface chemical states. The Zn 2p and Fe 2p signals remained (Figure 9b,c), indicating that Zn and Fe components persisted after five cycles. The Zn 2p_3_/_2_ and Zn 2p_1_/_2_ peaks were found at about 1020.65 and 1043.89 eV, and the Fe 2p_3_/_2_ and Fe 2p_1_/_2_ signals appear at approximately 710.50 and 724.09 eV, respectively, accompanied by an Fe(III)-related satellite peak at approximately 716.98 eV. In the Cu 2p region, the Cu 2p_3_/_2_ and Cu 2p_1_/_2_ peaks were seen at about 932.22 and 952.17 eV (Figure 9d). These lower Cu 2p binding energies match the presence of reduced Cu species on the catalyst surface after repeated exposure to NaBH_4_. However, because the Cu 2p binding-energy regions for Cu(I) and Cu(0) overlap, it is not possible to clearly identify the post-reaction Cu species as either Cu_2_O or metallic Cu based only on the Cu 2p spectrum. Overall, the XRD and XPS results after the reaction show that the main crystalline framework remains stable after five cycles, while the Cu-containing surface changes chemically under reaction conditions involving NaBH_4_. Together with AAS evidence of metal leaching, these results suggest that improving the structural and chemical stability of the CuO/ZnFe_2_O_4_ heterostructure, especially at the CuO-ZnFe_2_O_4_ interface, is an important area for future work.

According to Table 1, the CuO/ZnFe_2_O_4_ system achieves a significantly shorter reaction time of only 20 min, demonstrating rapid reduction under near-neutral conditions. Photocatalytic systems like Ce(MoO_4_)_2_/graphene oxide (GO) and Ag/TiO_2_ require longer reaction times and depend on external light irradiation. MOF-based (Al-MIL) and nano zero-valent iron (nZVI)/GO systems, though efficient, often need higher catalyst dosages or extended processing times. CuO/ZnFe_2_O_4_ stands out due to its fast kinetics, near-neutral pH operation, and operation without controlled external light irradiation, making it promising for practical NaBH_4_-assisted reduction. The initial CAP concentration of 12 mg·L^−1^ used in this study is much higher than that typically found in aquatic environments, where levels are typically in the nanogram per liter to low-microgram per liter range [33]. Therefore, further studies using environmentally relevant CAP concentrations are required to more comprehensively assess the practical applicability of the CuO/ ZnFe_2_O_4_ catalytic system.

**Table 1:** Comparison of CAP removal using different catalytic systems.

Material	Conditions	Catalytic	Light source	Reference

Ce(MoO_4_)_2_/GO	pH 5; *C*_CAP_ = 20 mg·L^−1^; *t* = 50 min	photocatalytic	visible	[34]
Ag/TiO_2_	pH 6.5; *C*_CAP_ = 20 mg·L^−1^; *t* = 120 min	photocatalytic	UV-C	[2]
persulfate	pH 3; *C*_CAP_ = 0.03 mM; *t* = 120 min	UV-activated persulfate oxidation	UV	[35]
Al-MIL	pH 5.4; *C*_CAP_ = 80 mg·L^−1^; *t* = 120 min	persulfate oxidation	–	[36]
nZVI/GO	pH 5; *C*_CAP_ = 100 mg·L^−1^; *t* = 180 min	photocatalytic	–	[37]
CuO/ZnFe_2_O_4_	pH 6; *C*_CAP_ = 12 mg·L^−1^; *t* = 20 min	NaBH_4_ reduction	–	this study

### Mechanism

**Scheme 1:**
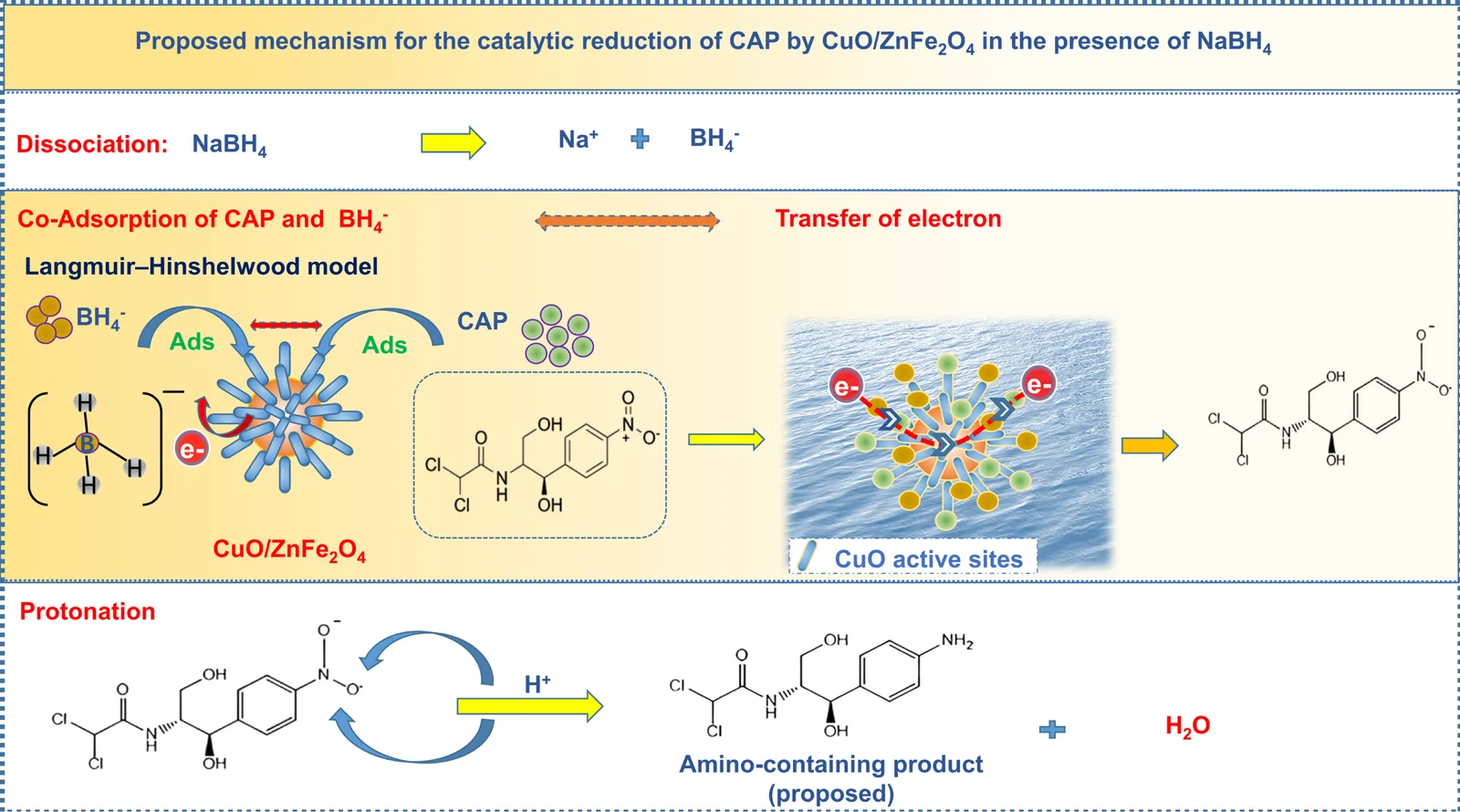
Proposed mechanism of catalytic CAP reduction by CuO/ZnFe_2_O_4_ in the presence of NaBH_4_.

Previous studies have demonstrated that aromatic nitro compounds can be reduced to their corresponding amino derivatives using NaBH_4_ [38–39]. Based on these findings, Scheme 1 illustrates the proposed CAP reduction pathway on the CuO/ZnFe_2_O_4_ heterostructure. Initially, NaBH_4_ dissociates in aqueous solution to release BH_4_^−^ ions, which may interact with the catalyst surface and facilitate electron transfer and the formation of reactive hydrogen species involved in CAP reduction. Simultaneously, CAP molecules are adsorbed onto the CuO/ZnFe_2_O_4_ surface, which could facilitate electron transfer between interfaces and subsequent reduction processes. As illustrated in Scheme 1, the nitro group of the CAP is proposed to undergo stepwise electron/proton transfer, leading to the formation of products consistent with the corresponding amino derivative (–NH_2_) and H_2_O.

Kinetic analysis indicated pseudo-first-order behavior during the initial reaction stage and a tendency toward zeroth-order behavior at later times. This shift in apparent kinetics may reflect progressive changes in surface coverage by CAP, reaction intermediates, BH_4_^−^-derived species, or borate products. The observations are qualitatively consistent with a surface-mediated Langmuir–Hinshelwood-type process in which adsorption and interfacial electron transfer contribute to CAP reduction; however, the kinetic data alone do not establish a unique mechanistic pathway. Overall, the CuO/ZnFe_2_O_4_ heterostructure provides favorable interfacial sites for adsorption and electron transfer in the NaBH_4_-assisted catalytic system. The proposed pathway is consistent with previously reported catalytic reduction processes for nitro-containing organic compounds [40].

## Conclusion

A CuO/ZnFe_2_O_4_ heterostructure was synthesized with a flower-like morphology composed of rod-like surface features associated with the CuO-containing component on the ZnFe_2_O_4_ surface. In the presence of NaBH_4_, the CuO/ZnFe_2_O_4_ system displayed markedly enhanced catalytic reduction efficiency for CAP compared to pristine ZnFe_2_O_4_, reaching maximum performance at pH 6, a catalyst loading of 2.5 g·L^−1^, and a reaction period of 20 min. The enhanced catalytic performance is attributed to the formation of the CuO/ZnFe_2_O_4_ heterostructure, which provides CuO-containing active sites and favorable interfacial interactions for reduction. Kinetic analysis indicated a change in apparent kinetic behavior during the reaction, with pseudo-first-order behavior in the initial stage and a tendency toward zeroth-order behavior at later times. This evolution is consistent with surface-mediated processes, although the present kinetic data do not establish a unique Langmuir–Hinshelwood mechanism. Reusability tests showed that CAP removal decreased only modestly from 91.0% in the first cycle to 86.8% after the fifth cycle. Post-reaction XRD showed that the major crystalline features of the heterostructure remained detectable after five cycles, while XPS indicated the presence of reduced Cu surface species, consistent with modification of the Cu-containing surface during repeated exposure to NaBH_4_. AAS analysis confirmed measurable Fe, Cu, and Zn leaching during repeated operation, identifying metal retention as a limitation of the present catalyst system. Overall, the CuO/ZnFe_2_O_4_ heterostructure demonstrates rapid NaBH_4_-assisted CAP reduction and substantial activity retention under simulated-wastewater and repeated-use conditions. Further development should focus on improving the heterostructure’s structural and chemical stability, particularly by strengthening the CuO–ZnFe_2_O_4_ interfacial interaction and suppressing metal leaching while preserving catalytic activity.

## Supporting Information

File 1 AAS measurements of dissolved metal ions.

## Data Availability

Data generated and analyzed during this study is available from the corresponding author upon reasonable request.
