# Supplementary material for: A CuO/ZnFe2O4 heterostructure as an efficient catalyst for NaBH4-assisted reduction of chloramphenicol
Source: Beilstein J Nanotechnol. 2026 Oct 16;17:1396–409. doi: 10.3762/bjnano.17.97 (PMC13678687; doi:10.3762/bjnano.17.97)
Supplement: Supplementary file 1 [file Beilstein_J_Nanotechnol-17-1396-s001.pdf]

BEILSTEIN JOURNAL OF NANOTECHNOLOGY

Supporting Information

for

A $CuO/ZnFe_2O_4$ heterostructure as an efficient catalyst for $NaBH_4$-assisted reduction of chloramphenicol

Le Thi Hoa, Nguyen Thi Thu Huong, Le Quoc Viet, Trinh Ngoc Dat, Nguyen Duc Cuong, Le Thi Thanh Nhi and Tran Thai Hoa

Beilstein J. Nanotechnol. **2026,** *17,* 1396–1409. doi:10.3762/bjnano.17.97

AAS measurements of dissolved metal ions

License and Terms: This is a supporting information file under the terms of the Creative Commons Attribution License (https://creativecommons.org/licenses/by/4.0). Please note that the reuse, redistribution and reproduction in particular requires that the author(s) and source are credited and that individual graphics may be subject to special legal provisions.
The license is subject to the *Beilstein Journal of Nanotechnology* terms and conditions: (https://www.beilstein-journals.org/bjnano/terms)

Table S1: Dissolved metal concentrations measured by AAS after each catalytic cycle.

Cycle	Fe (mg/L)	Cu (mg/L)	Zn (mg/L)
1	11.90	1.55	0.98
2	4.30	1.33	1.06
3	4.00	1.31	0.78
4	4.54	1.40	0.90
5	7.24	2.49	1.12

STT (GGNM)	*Tên (ký hiệu) mẫu*	*Chỉ tiêu TN*	*Phương pháp thử*	*V (ml)*	*Vdm (ml)*	*K*	*Cm (mg/L)*	*Ct (mg/L)*	*Xtb (mg/L)*	*Xtb (mg/L)*
		Fe	AOAC 999.11							
1	C12			1.7419	25	1	0.8307	0.00	11.9	**11.9**
2	C22			1.7262	25	1	0.2968	0.00	4.30	**4.30**
3	C32			1.6480	25	1	0.2634	0.00	4.00	**4.00**
4	C42			1.6414	25	1	0.2984	0.00	4.54	**4.54**
5	C52			1.7396	25	1	0.5037	0.00	7.24	**7.24**
		Cu	AOAC 999.11							
1	C12			1.7419	25	1	0.1082	0.00	1.55	**1.55**
2	C22			1.7262	25	1	0.0920	0.00	1.33	**1.33**
3	C32			1.6480	25	1	0.0865	0.00	1.31	**1.31**
4	C42			1.6414	25	1	0.0921	0.00	1.40	**1.40**
5	C52			1.7396	25	1	0.1732	0.00	2.49	**2.49**
		Zn	AOAC 999.11							
1	C12			1.7419	25	1	0.0684	0.00	0.98	**0.98**
2	C22			1.7262	25	1	0.0732	0.00	1.06	**1.06**
3	C32			1.6480	25	1	0.0511	0.00	0.78	**0.78**
4	C42			1.6414	25	1	0.0588	0.00	0.90	**0.90**
5	C52			1.7396	25	1	0.0778	0.00	1.12	**1.12**
